# The Utility of Social Media in Providing Information on Zika Virus

**DOI:** 10.7759/cureus.1792

**Published:** 2017-10-23

**Authors:** Neeraja Chandrasekaran, Kimberly Gressick, Vivek Singh, Jaclyn Kwal, Natalia Cap, Tulay Koru-Sengul, Christine L Curry

**Affiliations:** 1 Public Health Sciences, University of Miami Miller School of Medicine; 2 University of Miami Miller School of Medicine; 4 Department of Ob/gyn, University of Miami Miller School of Medicine

**Keywords:** public health, zika virus, social media

## Abstract

Introduction

In 2015, there was an outbreak of Zika virus in Brazil that spread throughout the Americas. The association of Zika virus with birth defects in infants born to infected pregnant women created concern for women of childbearing age. Social media is an important platform for health promotion, communication, and education on preventative methods during Zika virus outbreaks.

Methods

We evaluated the utility of social media on providing information regarding Zika virus. Facebook, Instagram, Twitter, and YouTube were utilized for our study. A search of the term “#Zikavirus” on Twitter and Instagram, and “Zika virus” on Facebook and YouTube was performed. The first 50 search results were analyzed from each source. Only English, Spanish, or Portuguese results were included. Results were categorized into three groups: “Useful”, “Not Useful”, or “Misleading”.

Results

Search was conducted on December 17th, 2016, with 185 results. Forty (21.6%) were from Facebook, 50 (27%) from Twitter, 48 (25.9%) from YouTube, and 47 (25.4%) from Instagram. A total of 104 (56.22%) results were "Useful", 67 (36.2%) "Not Useful", and 14 (7.5%) were "Misleading”. There were significantly more “Useful” results compared to “Not Useful” and “Misleading” results (Fisher’s exact: p < 0.0001).

Conclusion

Social media is a useful resource for providing relevant information on Zika virus. Young women can utilize social media for Zika virus information. The role of social media in public health should be further investigated and established. Patient education interventions should focus on social media impact on behavior modification and education of public to recognize useful information.

## Introduction

The outbreak of the Zika virus has brought about a significant global concern. Several cases of Guillain-Barre syndrome have been reported among adult population in Polynesia in 2014 that were found to be associated with Zika virus infection [1]. However, the link between birth defects and pregnancy suggested from current outbreak has created a major alarm for women of childbearing age [1, 2]. This association led to a rapid generation of Internet Google searches and tweets, which circulated within Brazil, Guatemala, and the United States from January 1 to February 29, 2016 [3]. New research about Zika is published on a frequent basis, as the information remains unknown. This rapid turnover of information poses difficulties to those responsible for educating the public. The non-traditional channels, like social media, can be used to address the increasing needs of rapid dissemination of information.

In recent years, social media has become a primary source of news and health information for many. According to a survey from the Pew Health Research Institute, 33% of United States consumers utilize social media search engines like Facebook, Twitter, and YouTube for healthcare information. Additional circulated information includes research articles, symptoms, offering opinions about doctors, treatments, drugs, and health plans [4]. In 2012, one billion people around the world were members of Facebook [4, 5]. In the medical field, the use of social media for circulating information and education doubled from 41% in 2010 to 90% in 2011 [5]. Ninety percent of medical students alone were found to use social media as an educational source [5, 6]. Moreover, the majority of people who obtain news from social media are women between the ages of 30-49 in the United States [4]. In 2016, Facebook and Instagram were the most commonly used social media sources by women and adolescent females [7, 8].

Due to its frequent usage by young women, social media may help in disseminating recent evidence-based information on Zika infection and updated pregnancy and travel-related press releases and recommendations by the healthcare authorities to prevent further spread. In fact, social media also played an important role in circulating such information during the summer Olympics Rio Grande, Brazil in 2016 [9]. The use of social media may help to educate public on the importance of barrier contraception, use of mosquito repellants and nets, wearing protective clothing, and avoiding travel to affected areas, as knowledge of Zika virus is significantly lacking in women of childbearing age and pregnant women [10-12]. In this study, we aim to assess the utility of social media in providing useful, factual, and timely information regarding Zika virus.

## Materials and methods

The four most widely utilized social media sources that are also used in medical education, communication and qualitative research, which were Facebook, Twitter, YouTube, and Instagram, were included [13-16]. To prevent filtering of information, a brand new and anonymous account was created for the Facebook and Instagram searches. The general search engine without an account was used for Twitter and YouTube. The terms “Zika” and “virus” were used for Facebook and YouTube, as Sharma, et al. previously utilized this in a study in an analysis of posts on Facebook [17]. For Instagram and Twitter, the term "#ZikaVirus" was searched, as the hashtag (#) categorizes conversations related to the respective topic in these sources [18]. The top 50 search results were analyzed, reviewed, and grouped into categories (Table 1) as 91% of users do not search past the first page of search results and 50% do not get past the first three results on the first page [19]. The categories consisted of three groups including: "Useful", "Not Useful", and "Misleading". The "Useful" group was further subcategorized into "Clinical" and "Informative". The "Not useful" category was further subdivided into "Intervention", "Commercial", "Commentary", and "Related". The "Misleading" category consisted of results that gave false information about Zika virus.

**Table 1:** Social media search result categorization criteria.

Categories	Description
I. Useful	Evidence-based and/or informative results for symptomatology and management of Zika virus
Clinically relevant	Direct evidence-based resources results which are useful for clinicians in their practice
Informative	News headline and other sources with correct symptomatology and geographical information
II. Not useful	Results regarding Zika virus but not clinically relevant
Intervention	Interventions that are not evidence-based
Commercial	Promote and sell products to protect from Zika virus
Commentary	Opinion-based results
Related	Neither informative nor relevant
III. Misleading	False sources, tweets, posts, articles, etc.

On Facebook, the top public posts and regular public posts were searched, which included a total of 40 results. On Twitter, the top 50 tweets of the day were included. On YouTube and Instagram, the top 50 search results were included. All posts that were written in English, Spanish, and Portuguese, and all other languages were excluded. The websites were never refreshed after the search was made. A separate analysis for outdated results was also performed for results that were in the "Useful" category. Results were considered outdated if it was posted before July 1, 2016 or was providing relevant information that did not pertain to the updated guidelines. Statistical analysis was performed with SAS studio® v9.4 (SAS Institute Inc., Cary, NC, USA). Descriptive analysis was performed along with the test of association using chi-square test or Fisher’s exact test at the significance level of 0.05. Permission was granted by the Institutional Review Board to perform this study as a Non-Human Subjects Study.

## Results

**Figure 1:**
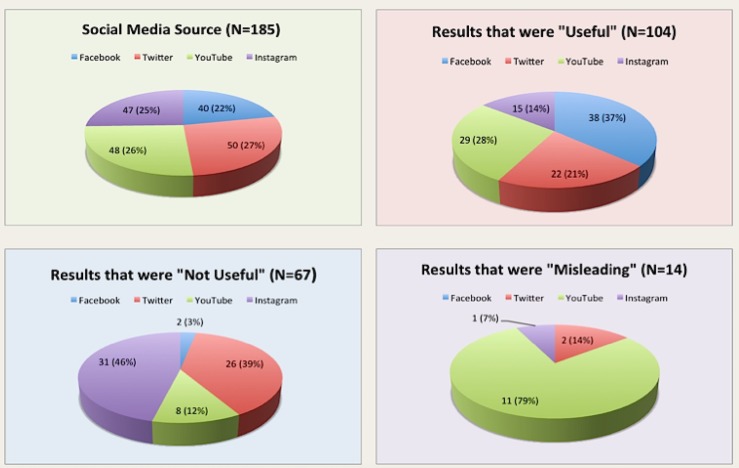
Social media sources and categorization.

The search and data collection was performed on December 17, 2016. A total of 185 search results were included in the analysis. There were two results from YouTube and three results from Instagram that were excluded, as they did not fit the language criteria. There were a total of 40 (21.6%) search results from Facebook, 50 (27.0%) from Twitter, 48 (25.9%) from YouTube, and 47 (25.4%) from Instagram (Figure 1). There were a total of 104 (56.2%) "Useful" results, 67 (36.2%) "Not Useful" results, and 14 (7.5%) "Misleading” results. There were significantly more “Useful” results compared to “Not Useful” and “Misleading” results (Fisher’s exact: p < 0.0001) (Figure 1).

**Figure 2:**
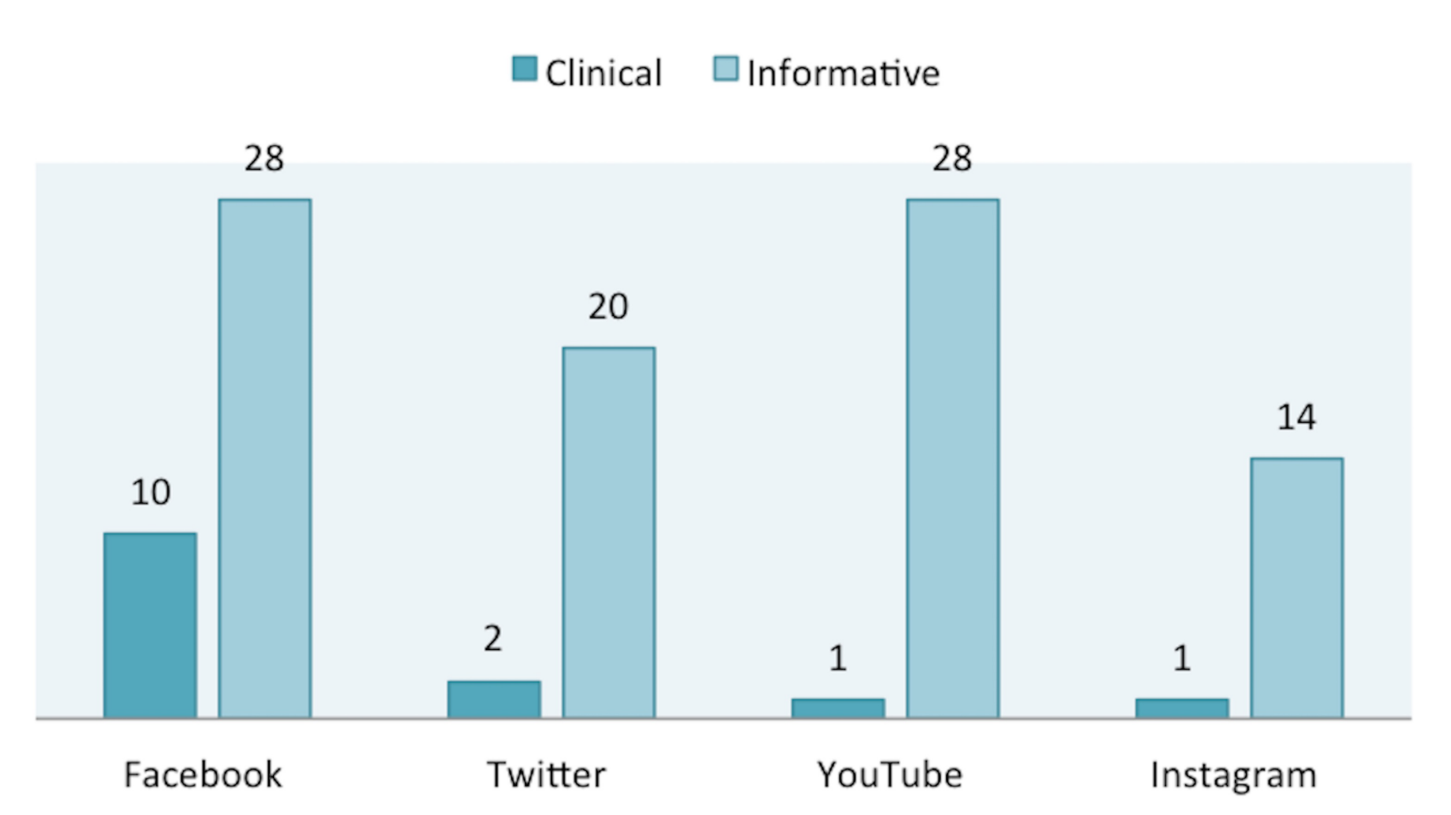
Subcategorization of "Useful" results.

**Figure 3:**
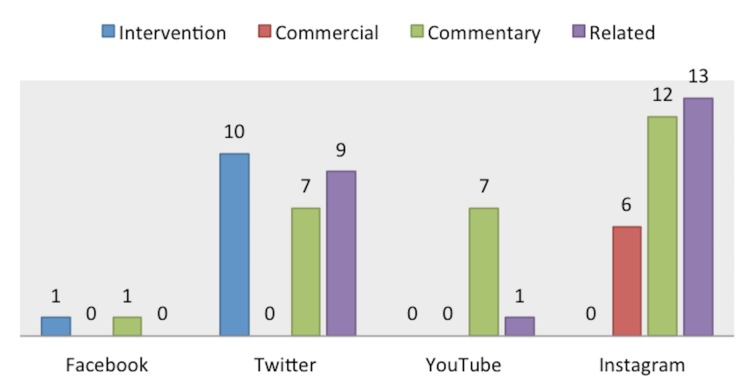
Subcategorization of "Not Useful" results.

Within the "Useful" category, 90 (86.5%) results were considered informative and 14 (13.4%) were considered clinically relevant (Figure 2). Facebook had the most clinically relevant information of 10 (71.4%) search results, and YouTube and Instagram had the least clinically relevant results of one (7.1%). Facebook and YouTube had the most informative results of 28 (31.1%). Instagram had the least informative information with 14 (15.5%) results. In the category of "Not Useful" search results, 11 (16.4%) were about an intervention, six (8.9%) were commercial results, 27 (40.3%) were considered commentary results, and 23 (34.3%) were related results (Figure 3). Twitter had the most posts for intervention results (10; 90.9%). Only Instagram had six (100%) commercial results. Instagram also had the most commentary (44.4%) and related (56.5%) results. Out of all the groups, YouTube contained the highest number of misleading results (78.5%). There were no misleading search results on Facebook.

There was a significant difference in outdated and current results amongst the social media groups in the “Useful” category (chi-square (3) = 40.40, p < 0.001). There were a total of 28 (26.92%) outdated results and 76 (73.08%) current results. Facebook had 30 current and eight outdated results. All the "Clinical" results were current, while eight "Informative" results were outdated. All 22 results on Twitter were current. YouTube had 20 outdated and nine current results. One "Clinical" and 19 "Informative" results were outdated. Only nine "Informative" results were current. There were no current "Clinical" results on YouTube. Instagram had a total of 15 current and no outdated results.

## Discussion

The outbreak of Zika virus has been a great source of global alarm, which has led to a rapid production and circulation of information to educate users and followers of social media. The top four most utilized social media sources include Facebook, YouTube, Instagram, and Twitter [13]. In this study, Facebook had the highest number of "Useful" articles with the most clinically relevant for medical providers compared to other social media groups. However, the generalizability for all searches in the future is unpredictable, as people may not be searching the top posts alone and misleading information has been found in prior studies. Sharma, et al. found that Facebook harbored misleading information regarding Zika virus in the week of June 21, 2016 [13]. Of the 200 posts, 12% (N = 21) were considered misleading [17, 20]. In contrast, there were no misleading search results in Facebook in our study. In addition, our study analyzed the search results only and we did not include group posts and shares.

Twitter is known for rapid turnover of tweets containing opinions and information. In medicine, it has been used for communication, updating information, and qualitative research [16, 21]. The use of Twitter in academic medicine for teaching and communication amongst medical students has also been very pronounced [15-17, 21]. In our study, there were more results that were "Not Useful" compared to "Useful". Of the "Useful" results, only two results were classified as "Clinical". Over the duration of one year, Fu, et al. observed a rapid rise in the weekly incidence of tweets regarding the impact of Zika virus, reaction to Zika virus, pregnancy and microcephaly, routes of transmission, and case reports [23]. In that duration, there were a significant amount of tweets with suspected cases, but with only a few confirmed cases of Zika virus [22].

In medicine, Instagram has also been utilized for educational and motivational purposes [23]. Notable uses for sharing of photos and videos have served more in fields like dermatology, infectious disease, and radiology [23]. In our study, Instagram had the most results that were “Not Useful” compared to the other social media sources. Most results, consisted of photographs, were opinions, commentaries, and messages promoting protection from Zika virus by using condoms and insect repellents. Although not widely utilized for medical information, Instagram may have the potential for educating female teenagers, since many adolescent females utilize Instagram [7].

The use of YouTube in healthcare has been controversial [24, 25]. Although it is an easily accessible source, it has been found to contain false medical information and the information about drugs and therapies that have not yet been approved by agencies like the Food and Drug Administration (FDA) [24-26]. In our study, YouTube had the highest number of misleading results consisted largely of videos posted by YouTube channels, members of YouTube, and news channels that stated relevant clinical information. This included hoax messages and conspiracy theories regarding Zika virus [25, 26]. In contrast, during the Ebola epidemic, Nagpal, et al. performed a study evaluating the first 100 search results on YouTube and found that YouTube videos that presented clinical symptoms of infectious disease during epidemics have increased odds of being more relevant (OR: 1.86, 95% CI: 1.06-3.28, p = 0.03) [26].

Our search yielded many outdated results, which YouTube had the most [6]. This may be because the videos posted on YouTube are mostly from groups and YouTube members that periodically post news on Zika. Therefore, there is not a rapid turnover of information on YouTube. Twitter and Instagram did not have any outdated information, which is likely because information is constantly updated and circulated on both media sources. Although Facebook had the most amount of "Clinical" results, most of the outdated results consisted of "Informative" results. It is important to provide patients with information on current guidelines while counseling as patients may receive outdated information from social media.

In this study, we captured and analyzed the top 50 results during a single time. However, the unpredictable nature of social media makes this hard to generalize to daily search results. Many adult and teenage women utilize social media [11, 12]. Thus, it may help to address barriers in preventative knowledge through education and communication. Physicians should always inquire level of knowledge and preventative methods from patients and ensure they receive correct information regarding Zika virus. Physicians should also directly utilize evidence-based resources for clinical information, as social media does not contain much evidence-based information. The role of social media in promoting health information and communication during disease outbreaks should be further investigated. In addition, interventions should focus on educating public to be able to recognize useful information.

## Conclusions

Social media is a useful medium for pregnant women and women of childbearing age to access information regarding Zika virus. Despite the usefulness of social media, it is important for clinicians to provide recent evidence-based information during patient encounter, as some sources on social media are outdated. The use of social media in public health and providing information during disease outbreaks should be further established.
